# Influence of Hematocrit and Storage Temperature on the Stability of Dried Blood Samples in Serological Analyses of Tetanus, Diphtheria, and Pertussis

**DOI:** 10.3390/diagnostics14192195

**Published:** 2024-10-01

**Authors:** Mariano Rodríguez-Mateos, Silvia Carlos, Javier Jaso, África Holguín, Gabriel Reina

**Affiliations:** 1 Microbiology Department, Clínica Universidad de Navarra, 31008 Pamplona, Spain; mrodriguezm@unav.es (M.R.-M.); jjaso@alumni.unav.es (J.J.); 2 Department of Preventive Medicine and Public Health, Universidad de Navarra, 31008 Pamplona, Spain; 3 IdiSNA, Navarra Institute for Health Research, 31008 Pamplona, Spain; 4 HIV-1 Molecular Epidemiology Laboratory, Microbiology and Parasitology Department, University Hospital Ramón y Cajal-IRYCIS and CIBEREsp-RITIP, 28034 Madrid, Spain; africa.holguin@salud.madrid.org

**Keywords:** dried blood spots (DBSs), serology, chemiluminescence, immunoassay, diphtheria, tetanus, pertussis

## Abstract

Background: Dried blood spots (DBSs) enable the study of serological markers of various pathogens without the need to obtain serum/plasma through venipuncture. Methods: Sixty-four blood samples were prepared on Whatman™ 903 cards using specimens obtained by venipuncture to study the detection of serological markers of diphtheria, tetanus, and pertussis in DBSs, and their stability 4 months post-collection. An automated chemiluminescent immunoassay was used to investigate diphtheria, tetanus, and pertussis IgG levels from both DBSs and plasma samples. Results: An optimal cut-off value for DBSs was calculated to improve the performance of diphtheria and tetanus serological markers in DBSs, achieving high sensitivity (95% and 98%, respectively) and specificity (91.7% and 92.3%, respectively). No protection against pertussis was found in the population studied. The correlation observed between the plasma and the DBSs processed after sample collection was high (0.967–0.970) for all antibodies studied except pertussis (0.753), both considering hematocrit before sample elution or not. The correlation between DBSs and plasma for diphtheria and tetanus remained strong following a 4-month delay in DBS processing at 4 °C (0.925–0.964) and −20 °C (0.924–0.966), with only a minor decrease observed for diphtheria at room temperature (0.889), while maintaining a strong correlation for tetanus (0.960). For pertussis, the correlation between DBSs and plasma was drastically reduced after delaying its processing for 4 months at any temperature. Conclusions: To summarize, hematocrit levels within the normal range do not affect the processing of DBSs in the study of serological markers of diphtheria, tetanus, and pertussis. The DBS stability for serological diagnosis of diphtheria and tetanus is adequate when samples are stored at −20 °C for a period of 4 months. The pertussis serological marker does not appear to remain stable after 4 months, even when the DBS is stored frozen at −20 °C.

## 1. Introduction

Dried blood spots (DBSs) are a sampling technique which works with a small quantity of blood applied onto filter paper, left to air-dry at ambient temperature for several hours, and are easily stored thereafter. Numerous studies have demonstrated the utility of DBS in serological testing by using it to monitor various diseases, thanks to its precise detection of antibodies compared to serum/plasma [1,2,3,4,5,6,7,8].

In low- and middle-income nations, DBSs offer an economical, non-invasive solution for diagnostic testing, requiring minimal resources and expertise compared to venipuncture [9,10]. In high-income settings, DBSs facilitate outreach to individuals disconnected from healthcare systems, enhancing patient and participant engagement with healthcare services [11,12]. Additionally, the minimal blood volume requirement is particularly advantageous for pediatric diagnostics [13]. Moreover, DBSs offer simplified storage and transport compared to serum/plasma, occupying less space and weight and avoiding classification as infectious material [14]. Recognizing these advantages, the World Health Organization (WHO) endorses DBS adoption as a viable alternative to venipuncture [15].

Despite the DBS’s widespread use in various serological studies, questions persist about their reliability in analyzing additional biomarkers, such as those for vaccine-preventable diseases like diphtheria, tetanus, and pertussis. Successful validation of this method could notably advance the diagnosis and surveillance of these infectious illnesses. Variability in the correlation between serum/plasma and DBS results has been noted across different pathogens, suggesting potential differences in antibody stability within DBSs [6,16]. Furthermore, there are currently no established guidelines for the storage of DBSs to preserve antibody stability, underscoring the importance of ensuring proper processing and storage conditions to facilitate accurate analyses.

Diphtheria, tetanus and pertussis are common and serious bacterial vaccine-preventable diseases worldwide. Regardless of the availability of highly effective vaccines, these bacterial infections remain a public health concern in several countries, both in resource-rich and resource-limited [17,18,19,20,21]. Understanding the seroprevalence of these diseases is crucial for assessing the effectiveness of vaccination strategies and guiding public health policies aimed at controlling and preventing them.

Our objective was to assess the capacity of DBSs in the detection of serological markers of diphtheria, tetanus, and pertussis using an indirect chemiluminescent immunoassay (CLIA). We also examined the impact of hematocrit levels on the markers when using DBSs. Additionally, we explored the influence of storage temperature on the stability of these markers within DBSs.

## 2. Materials and Methods

### 2.1. Study Design and Participants

A serological sectional study was conducted from October to December 2019. Blood samples from 64 adult patients visiting Clínica Universidad de Navarra (Pamplona, Spain) for routine analysis were collected. All participants were over 18 years old, selected to ensure a proportional representation of both sexes, different age groups, and hematocrit levels. Pregnant women and individuals with autoimmune diseases or infectious mononucleosis were excluded due to the elevated probability of false-positive results in serological analyses. Patient identities were coded during sampling to safeguard confidentiality.

### 2.2. Sample Collection and Storage

A sample of anticoagulated blood with EDTA was collected from each patient via venipuncture. The hematocrit of all blood samples was measured, together with a complete blood count. Subsequently, five DBSs were prepared for each patient by inoculating 70 μL of whole blood per DBS onto Whatman™ 903 cards (GE Healthcare, Chicago, IL, USA) and drying at room temperature for 24 h. The remaining blood volume was processed to obtain plasma by centrifuging at 2000× *g* for ten minutes. Once dry, all DBSs were cut with scissors and placed into individual Eppendorf microtubes. For each patient, two DBSs (DBS-A and DBS-B) were processed immediately after collection, while the remaining three DBSs were stored at different temperatures to assess their stability over a 4-month period before analysis (at −20 °C (DBS-C), at 4 °C (DBS-D) and at room temperature (DBS-E)).

### 2.3. DBS Elution

**Figure 1:**
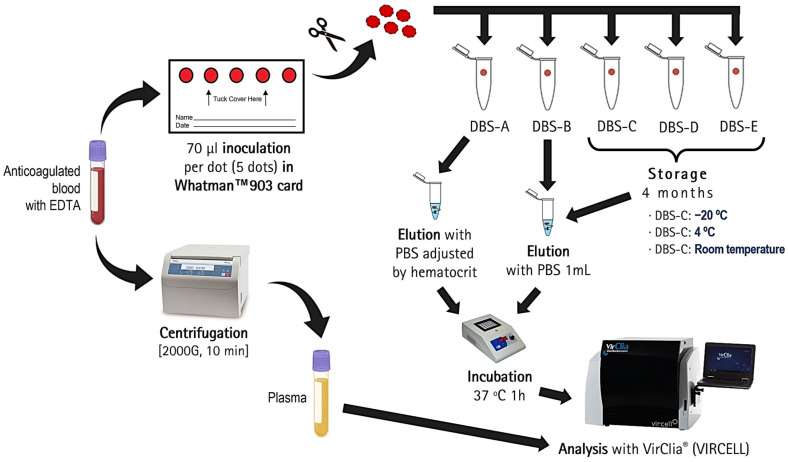
Processing and storage conditions of DBSs and plasma for serological diagnosis. PBS: phosphate-buffered saline; DBS-A: DBS processed immediately and eluted by adjusting the volume of PBS taking the hematocrit value into account; DBS-B: DBS immediately and eluted with 1 mL of PBS regardless of the hematocrit; DBS-C: DBS stored at −20 °C for 4 months; DBS-D: DBS stored at 4 °C for 4 months; DBS-E: DBS stored at room temperature for 4 months.

One DBS (DBS-A) was eluted by adjusting the volume of phosphate-buffered saline (PBS) according to the hematocrit to ensure an exact equivalence with the volume of plasma used in standard assays (5 µL/assay) (Figure 1). The other four DBSs (DBS-B, DBS-C, DBS-D and DBS-E) were eluted with 1 mL of PBS regardless of the hematocrit. To carry out the elution, all five DBSs were then incubated at 37 °C for 1 h. To enhance elution, the tubes were vortexed every 15 min. Once elution was complete, the residual paper was removed to prevent instrument obstruction.

### 2.4. Serological Testing

The VirClia^®^ automated system (Vircell, Granada, Spain) was used to perform the detection of IgG against *Corynebacterium diphtheriae*, *Clostridium tetani*, and *Bordetella pertussis* toxins by indirect chemiluminescent immunoassay (CLIA), both in DBSs and plasma. The protocols employed for conducting chemiluminescence tests using DBSs differed slightly from that utilized with plasma samples, involving adjustments in sample volume. Specifically, 105 microliters of eluted DBSs were introduced into the sample well for DBS testing, whereas plasma sample diagnosis entailed the use of 5 μL of a sample diluted with 100 microliters of diluent (1/21 dilution).

The results were interpreted in accordance with the manufacturer’s instructions, considering the plasma cut-off values provided as the gold standard (96–100% sensitivity and 100% specificity).

### 2.5. Statistical Analysis

A comparison of assay results between DBSs and plasma was conducted, and correlation was evaluated using Spearman’s test, given the non-normal distribution of the samples determined with the Shapiro–Wilks test. The level of agreement between the two DBS elution methods was determined using the kappa coefficient. To enhance the utility of DBSs for IgG measurements, sensitivity, specificity, positive predictive values (PPVs), and negative predictive values (NPVs) were calculated for each parameter, with plasma results serving as the gold standard. Optimal cut-off indices for interpreting DBS results for each parameter were determined by calculating the area under the curve of the receiver operating characteristics (ROC) curves. Microsoft Excel version 2021 and Stata 15 were used to calculate statistics and generate graphs. Differences between groups were studied using the Chi-square test. Statistically significant differences were considered when the *p*-value was below 0.05.

## 3. Results

The general characteristics of the study population are shown in Table 1. The median age of the individuals studied was 42.6 years, and 90.6% were born in Spain.

### 3.1. Immunization Levels in Plasma

Protective IgG levels varied for each disease in the study cohort. We found that the level of protection against diphtheria was moderate (61.9%), high for tetanus (79%), and absent (0%) for pertussis (Table 1). The protection levels against diphtheria and tetanus exhibited a trend of declining with age.

For diphtheria, a notable discrepancy in protection is observed across age groups, with a higher rate among younger individuals and a significant decrease as age increases. Specifically, among those under 30 years old, a 100% protection rate is recorded for both males and females, whereas in the population group aged 60 years and above, protection decreases significantly to only 30.8% in the overall population. A similar trend is observed for tetanus, where protection levels are also higher in younger groups and diminish in older age groups. The protection coverage rate was higher in women than in men, with statistically significant differences observed in the case of tetanus, particularly in the older population group, where men exhibited a protection rate of only 14.3% compared to 50% in women (*p* = 0.046). In the case of diphtheria, statistically significant differences between males and females were only observed in the population group aged 60 years or older, where a protection rate of 0% was observed for males compared to 66.7% for females (*p* = 0.002). No inconclusive results were observed for diphtheria and tetanus; however, 8.8% of patients exhibited indeterminate results for pertussis.

### 3.2. Correlation between DBSs and Plasma

**Figure 2:**
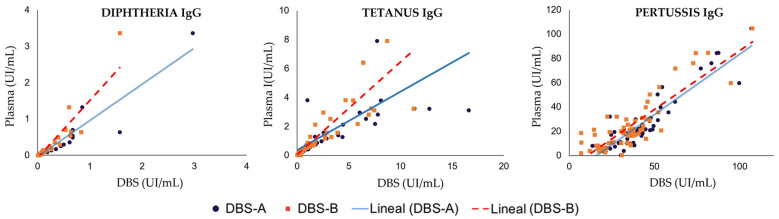
Comparison of DBS and plasma results for diphtheria, tetanus, and pertussis, processed immediately following sample collection. DBS-A: DBS eluted with a volume of PBS considering hematocrit; DBS-B: DBS eluted with 1 mL of PBS regardless of hematocrit.

**Figure 3:**
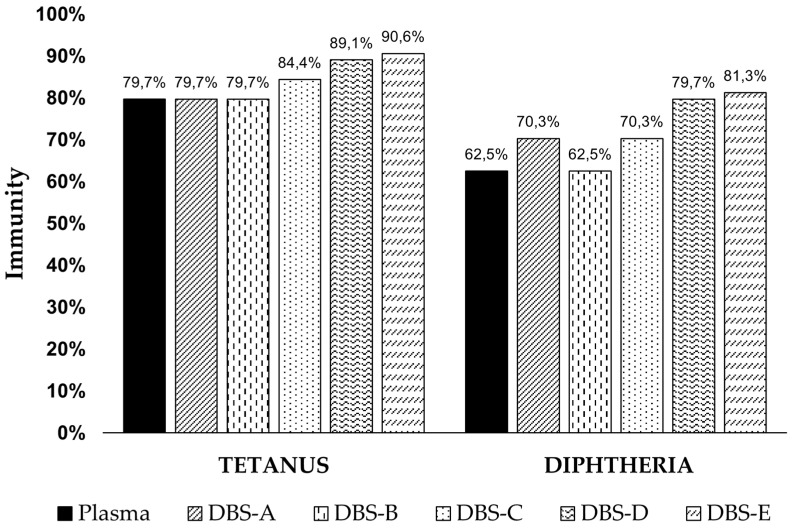
Immunity (IgG) against diphtheria and tetanus in our study population calculated using the manufacturer cut-off values for plasma (>0.01 IU/mL for diphtheria and >0.1 IU/mL for tetanus) and the optimized DBS cut-off values for all DBS analysis (>0.028 IU/mL for diphtheria and >0.243 IU/mL for tetanus). DBS-A: DBS processed immediately and eluted with a volume of PBS based on the hematocrit; DBS-B: DBS processed immediately and eluted with 1 mL of PBS; DBS-C: DBS stored at −20 °C for 4 months; DBS-D: DBS stored at 4 °C for 4 months; DBS-E: DBS stored at room temperature for 4 months.

The correlation analysis between DBS and plasma results was strong for diphtheria and tetanus serology, with Spearman’s correlation coefficients ranging from 0.961 to 0.966 and 0.971 to 0.983, respectively (Figure 2, Table 2). Although pertussis also showed a significant strong correlation (0.753), it was comparatively weaker. However, quantitative outcomes obtained via immunoassay using DBS samples were marginally elevated compared to those from plasma samples, resulting in compromised specificity when interpreting DBS results based on manufacturer-established plasma cut-off values (0.01 IU/mL for diphtheria and 0.1 IU/mL for tetanus). Application of these plasma cut-offs to DBS interpretation led to a higher percentage of immunized patients for all pathogens compared to plasma analysis, with specificity reduced to only 4.2% for diphtheria and 53.8% for tetanus. Consequently, novel optimized DBS cut-off values were calculated for each target utilizing ROC curves to achieve optimal sensitivity and/or specificity in IgG detection using DBSs. These optimized cut-off values were determined to be 0.028 IU/mL for diphtheria and 0.243 IU/mL for tetanus. The implementation of new optimized cut-offs for DBSs reduced the percentage of immunized patients for both diphtheria and tetanus compared to the results obtained using plasma cut-off, although these percentages remained slightly higher than those observed in plasma samples when the processing of the DBSs was delayed for 4 months (Figure 3). Notably, utilization of these new DBS cut-offs enhanced the specificity, positive predictive value (PPV), and negative predictive value (NPV) of the tests (Table 3).

#### 3.2.1. Effect of the Hematocrit

The DBS samples eluted with a volume of PBS based on the hematocrit value (DBS-A) yielded very similar results to those obtained with DBSs eluted universally with 1 mL of PBS (DBS-B) for both diphtheria and tetanus serological markers (Figure 2). Only in the case of tetanus did the specificity decrease from 69.2% to 53.8% when the DBS was eluted without considering the hematocrit; however, this decrease in specificity disappears when the new cut-off for DBS samples was used. Comparing both elution methods to each other, we found excellent agreement between them, with kappa values of 1.00 for diphtheria and 0.85 for tetanus.

#### 3.2.2. DBS Stability

For both diphtheria and tetanus serological markers, delayed processing of DBS samples barely diminished their correlation with plasma. In the case of diphtheria, the correlation with plasma was reduced by 4.45% when the DBS sample was stored at −20 °C, 4.39% at 4 °C, and 8.10% at room temperature. Regarding tetanus, the reduction in correlation was slightly lower, decreasing by only 0.35% when the sample was stored at −20 °C, by 0.56% when stored at 4 °C, and by 1.47% when kept at room temperature. In contrast, the pertussis serology in DBSs exhibited a drastic drop in correlation with plasma when processing was delayed for four months, decreasing by 21.53% at −20 °C, 53.44% at 4 °C, and 78.50% at room temperature (Table 2). This decline in the correlation after four months of storage led to a decreased specificity for diphtheria and tetanus. However, due to the tendency for serological values obtained from DBSs to register higher levels compared to those from plasma, there was no impact on the sensitivity of the assays (Table 3).

## 4. Discussion

The use of DBSs represents a convenient tool for conducting seroprevalence studies, with significant potential to facilitate remote and decentralized testing, thereby contributing to improve accessibility and efficiency in healthcare delivery. Our study not only highlights the equivalence in detecting antibodies against diphtheria, tetanus, and pertussis using DBSs compared to plasma but also demonstrated that DBS samples stored for up to 4 months remain valid for studying diphtheria and tetanus serologies, even at room temperature, although this prolonged validity of the DBS sample does not seem to apply to pertussis analysis. Additionally, we have shown that eluting DBS samples does not need to be adjusted based on hematocrit and can be easily performed using a universal elution procedure without compromising analytical validity.

Our findings demonstrate that DBS samples facilitate the verification of previous exposure or immunization against diphtheria, tetanus, and pertussis, using an automated chemiluminescent immunoassay. In essence, this procedure enables the study of seroprevalence against these vaccine-preventable diseases, providing invaluable information to verify if herd immunity has been achieved within the community. Furthermore, we report optimized cut-off values for DBS specimens when employing the VirClia^®^ automated platform (Vircell) to detect IgG antibodies of diphtheria and tetanus, ensuring both high sensitivity and specificity. The optimal threshold values for assessing the results of chemiluminescence for each parameter were higher than the cut-off index applied to plasma samples (0.01 IU/mL for diphtheria and 0.1 IU/mL for tetanus), enhancing the interpretation of results obtained from DBSs in comparison to plasma, with high sensitivities ranging from 95 to 98% and specificities from 91.7 to 92.3%. However, it is important to acknowledge that establishing the optimal cut-off values ensuring heightened sensitivity and specificity for the identification of protective IgG against tetanus and diphtheria requires individual calculation within each study cohort utilizing paired plasma/DBS specimens. Subsequent extrapolation of such determinations is warranted solely to populations sharing analogous infection prevalence profiles. This limitation becomes evident when we observe that different optimal cut-offs have been calculated in other studies similar to ours conducted in other populations [22].

Both DBS samples processed with volume of elution adjusted according to hematocrit (DBS-A) and those processed without adjustment (DBS-B) exhibited comparable results to those obtained with plasma, achieving excellent correlation coefficients ranging from 0.961 to 0.983 for diphtheria and tetanus, and moderately lower coefficients of 0.753–0.849 for pertussis. This demonstrates that elution of DBSs for serological diagnosis can be effectively performed using a universally unadjusted elution volume, thereby greatly facilitating laboratory procedures. These findings are consequent with previous studies that observed similar results with other serological markers such as Epstein–Barr virus [23], hepatitis E [24], and Chikungunya virus [25], as well as our previous report on measles, mumps, and rubella [6], and even other analytes (creatinine, testosterone, and drug monitoring) [26,27,28].

To assess the stability of DBS samples for serological analysis after prolonged storage (120 days), three different storage temperatures (−20 °C, 4 °C, and room temperature) were evaluated. The correlation with plasma results for diphtheria was excellent at −20 °C (0.921) and 4 °C (0.922), decreasing only slightly when the DBS sample was stored at room temperature (0.855). For tetanus, the correlation was excellent at any temperature, including room temperature (0.960–0.983). Conversely, delaying the processing of DBSs for 4 months drastically reduced the correlation between DBSs and plasma for the pertussis marker, with correlations of 0.591 at −20 °C, 0.351 at 4 °C, and only 0.162 at room temperature. Therefore, despite the significant loss of marker positivity observed in other serological assays when DBSs are not stored under cold conditions [6,16,29], DBS samples could be valid for at least 4 months for the serological study of diphtheria and tetanus even if not stored under controlled refrigeration conditions. However, despite the excellent correlation data, our findings indicate that DBS samples tend to show higher antibody concentrations for diphtheria and tetanus compared to paired plasma samples, with this discrepancy increasing during prolonged storage. This effect is more pronounced at higher storage temperatures, leading to an artificial elevation in the detected levels. The increased IgG signal observed after 4 months may be attributed to several technical factors: (a) over time, non-target proteins in the dried blood spots may degrade or change in conformation, leading to cross-reactivity with the detection antibodies used in the assay [30]; (b) prolonged storage can induce matrix effects, such as protein–protein interactions or the binding of proteins to the filter paper, which may release more IgG or other proteins upon extraction, contributing to non-specific signals [31]; and (c) stored IgG can undergo degradation and fragmentation over time, resulting in the formation of smaller fragments that may still react with detection antibodies, leading to falsely elevated non-specific signals [32]. As a result, this artificial increase in antibody concentrations contributes to a loss of specificity, making DBS samples unsuitable for studying diphtheria and tetanus markers when stored for 4 months at room temperature or even at 4 °C. However, DBS samples stored at −20 °C may still retain sufficient accuracy. Therefore, it may be necessary to establish new cut-off values for stored DBS samples to mitigate this loss of specificity. In the case of pertussis, the stability of antibodies against *B. pertussis* toxin in DBSs appears to be greatly affected over time even when the sample is frozen at −20 °C. Unfortunately, due to the lack of immunity observed for pertussis, these results are likely inconclusive and should be further studied in populations with higher antibody levels.

Systemic vaccination against diphtheria, tetanus, and pertussis was introduced in Spain in 1965 using an inactivated whole-cell pertussis vaccine combined with diphtheria and tetanus toxoids (DTPs). Since then, various modifications have been performed regarding its administration. The common childhood vaccination schedule approved by the Interterritorial Council of the National Health System in 2024 includes a scheme with an acellular pertussis vaccine combined with diphtheria and tetanus toxoids (DTaPs) administered at 2, 4, and 11 months of age, with a booster dose at 6 years, and a booster dose against diphtheria and tetanus at 14 years of age. Additionally, for the adequate protection of infants in the early months of life, the vaccination of pregnant women against pertussis is recommended starting from the 27th week of gestation, preferably in the 27th or 28th week, using a low-dose acellular pertussis vaccine (dTpa). Furthermore, individuals over 65 years of age are administered a booster dose against tetanus and diphtheria (Td) [33]. The vaccination coverage of DTaPs in Spain has remained consistently high over the past decades, exceeding 90% in children and 80% in pregnant women, according to data from the Vaccination Information System of the Ministry of Health [34]. These vaccine coverage data are consistent with the seroprevalence observed in our study for diphtheria and tetanus; however, despite high vaccination coverage, the seroprevalence of pertussis observed in our study was completely absent. Notably, the second seroprevalence study of vaccine-preventable diseases in Spain reported a low prevalence of pertussis antibodies, with only 1.6% of individuals testing positive out of a total of 6143 serum samples from individuals aged 2–80 years old [35]. This limited seroprevalence of pertussis antibodies in the Spanish population could explain the absence of pertussis seroprevalence observed in our study, given the limited sample size of our study. Moreover, the vaccine-induced immunity against pertussis diminishes within the first three years and disappears between 4 and 12 years after the fifth and final dose; conversely, naturally acquired immunity fades away between 4 and 20 years after contracting the disease [36,37,38]. Considering this, it is not surprising that by adulthood, immunity has waned, rendering individuals susceptible.

The interpretation of pertussis seroprevalence results is challenging due to the lack of a well-defined antibody threshold indicative of protection and the absence of a surrogate parameter for protection [39]. A previous study has demonstrated that antibody titers of at least 100 IU/mL correlate with recent infection within the past year by *B. pertussis*; such levels are present in less than 1% of the population and are reached in the majority of pertussis patients within four weeks after onset of illness, persisting only temporarily [40]. This would imply that there was no circulation of the microorganism in the period immediately preceding our study, which could explain our results.

In the case of immunity observed against diphtheria and tetanus, we generally found a higher rate of protection in women rather than in men, particularly among those above 60 years old. Previous studies have consistently shown that, overall, women have higher antibody titers than men, but immunity against diphtheria and tetanus can be higher in men [41,42]. This greater protection in men reported by other authors could be attributed to higher vaccination rates among men during military service and a higher frequency of booster doses following injuries, which may not be occurring in our study population.

Our research faces several potential limitations. First, the vaccination records of participants were unavailable, preventing us from gathering information about expected biomarkers. Additionally, the pre-infection rate remained undisclosed; nevertheless, the extensive vaccination coverage within our demographic may facilitate the evaluation of these antibodies. While the results are presented through data subgrouping based on age group and hematocrit levels, it is noteworthy that the study was conducted subsequently to the homogeneous selection of participants concerning age, sex, and hematocrit. While our study showed that hematocrit levels did not significantly impact the performance of DBSs in chemiluminescent assays, it is important to note that this conclusion is based primarily on samples with normal hematocrit levels. The influence of abnormal hematocrit levels on assay performance remains uncertain and warrants further investigation. Additionally, the DBSs were obtained through venipuncture rather than fingertip pricks, which are more commonly used in clinical settings. It is important to note that venipuncture and fingertip blood may differ in several components, such as cell counts and protein concentrations. These differences could potentially influence the results, particularly when interpreting cut-off values and antibody concentrations. Future studies should explore whether the findings obtained from venipuncture-derived DBSs are directly applicable to DBS samples collected from fingertip pricks in clinical practice. Lastly, we did not encounter any patients with immunity to pertussis in our population; thus, we were unable to thoroughly examine how the pertussis serological marker behaves in DBSs compared to plasma, or assess the impact of various storage conditions on this marker. Despite these limitations, our study exhibits several notable strengths, including the utilization of standardized, commercial and validated serological platforms, ensuring the robust measurement of pertinent values for the investigation.

## 5. Conclusions

Our findings confirm the reliability of DBS samples for studying serological markers of diphtheria and tetanus. Additionally, hematocrit levels within the normal range do not appear to have a significant impact on the performance of DBSs in chemiluminescent assays. DBSs are sufficiently stable for diphtheria and tetanus antibody testing when stored at −20 °C for a period of four months. However, the cut-off values should be optimized when using DBSs for seroprevalence studies.

Employing DBSs could enhance the detection of antibodies and, therefore, the monitoring of seroprotection levels of diphtheria and tetanus when collecting plasma/serum poses challenges or is unfeasible. While the pertussis serological marker does not seem to remain stable after 4 months, even when DBSs are stored frozen at −20 °C, we recommend conducting further studies with a larger sample size to elucidate the performance of these markers for pertussis in DBSs.

## Figures and Tables

**Table 1:** Characteristics of the study population according to age groups.

	Male	Female	Total
*n* (%)	31 (48%)	33 (52%)	64
Median age at sampling [IQR]	42.8 [37.6–58.3]	41.8 [31.6–56.7]	42.6 [36.5–58.1]
Age Groups			
18–30	5 (16%)	7 (21%)	12 (18.8%)
30–45	12 (39%)	12 (36%)	24 (37.5%)
45–60	7 (23%)	8 (24%)	15 (23.4%)
>60	7 (23%)	6 (18%)	13 (20.3%)
Median hematocrit by age group			
18–30	42.7	40.5	41.9
30–45	44.8	41.0	42.1
45–60	43.2	38.6	41.0
>60	38.2	38.1	38.2
Total [IQR]	42.7 [38.9–45.7]	39.8 [37.2–41.7]	41.0 [37.8–43.6]
Pathogen immunity (plasma), by age group			
Diphtheria			
18–30	100%	100%	100%
30–45	83.3%	75.0%	79.2%
45–60	42.9%	25.0%	33.3%
>60	0% *	66.7% *	30.8%
Total	58.1%	66.7%	62.5%
Tetanus			
18–30	100%	100%	100%
30–45	91.7%	100%	95.8%
45–60	71.4%	87.5%	80.0%
>60	14.3% *	50.0% *	30.8%
Total	71.0% *	87.9% *	79.7%
Pertussis	0%	0%	0%

IQR: interquartile range; age in years; Hematocrit in %. Immunity is defined as having IgG levels greater than 0.01 IU/mL for diphtheria, 0.1 IU/mL for tetanus, and >120 IU/mL for pertussis. IgG levels for pertussis between 60 and 120 IU/mL are classified as indeterminate. * Statistically significant differences (*p* < 0.05).

**Table 2:** Spearman’s correlation coefficients between DBS and plasma results.

	DBS-A (T_0_)	DBS-B (T_0_)	DBS-C (T_4m_, −20 °C)	DBS-D (T_4m_, 4 °C)	DBS-E (T_4m_, RT)
DIPHTHERIA	0.961	0.966	0.921 (−4.45%)	0.922 (−4.39%)	0.855 (−8.10%)
TETANUS	0.983	0.971	0.978 (−0.35%)	0.968 (−0.56%)	0.960 (−1.47%)
PERTUSSIS	0.849	0.753	0.591 (−21.53%)	0.351 (−53.44%)	0.162 (−78.50%)

DBS-A: DBS processed without delay (T_0_) and eluted with an adjusted volume of PBS, taking into account the hematocrit; DBS-B: DBS without delay and eluted with 1 mL of PBS; DBS-C: DBS stored at −20 °C for 4 months (T_4m_); DBS-D: DBS stored at 4 °C for 4 months (T_4m_); DBS-E: DBS stored at room temperature (RT) for 4 months (T_4m_). The loss of correlation between DBS-B and the DBS whose processing is delayed at 4 months (DBS-C, DBS-D, and DBS-E) is shown within parentheses.

**Table 3:** Results of VirClia^®^-IgG test for the detection of protective IgG against diphtheria and tetanus.

		Diphtheria *				Tetanus **			
		S	Sp	PPV	NPV	S	Sp	PPV	NPV
DBS-A (T_0_)	Plasma cut-off	100%	4.2%	63.5%	100%	100%	69.2%	92.7%	100%
	Optimized DBS cut-off	97.5%	75.0%	86.7%	94.7%	98.0%	92.3%	98.0%	92.3%
DBS-B (T_0_)	Plasma cut-off	100%	4.2%	63.5%	100%	100%	53.8%	89.5%	100%
	Optimized DBS cut-off	95.0%	91.7%	95.0%	91.7%	98.0%	92.3%	98.0%	92.3%
DBS-C (T_4m_, −20 °C)	Plasma cut-off	100%	4.2%	63.5%	100%	100%	23.1%	83.6%	100%
	Optimized DBS cut-off	92.5%	66.7%	82.2%	84.2%	100%	76.9%	94.4%	100%
DBS-D (T_4m_, 4 °C)	Plasma cut-off	100%	4.2%	63.5%	100%	100%	23.1%	83.6%	100%
	Optimized DBS cut-off	95.0%	45.8%	74.5%	84.6%	100%	53.8%	89.5%	100%
DBS-E (T_4m_, RT)	Plasma cut-off	100%	4.2%	63.5%	100%	100%	7.7%	81.0%	100%
	Optimized DBS cut-off	95.0%	41.7%	73.1%	83.3%	100%	46.2%	87.9%	100%

* Diphtheria cut-off, plasma: 0.01 IU/mL; optimized DBS cut-off: 0.028 IU/mL; ** Tetanus cut-off, plasma: 0.1 IU/mL; optimized DBS cut-off: 0.243 IU/mL. S: sensitivity; Sp: specificity; PPV: positive predictive value; NPV: negative predictive value. DBS-A: DBS processed without delay and eluted with an adjusted volume of PBS, taking into account the hematocrit; DBS-B: DBS without delay (T_0_) and eluted with 1 mL of PBS; DBS-C: DBS stored at −20 °C for 4 months (T_4m_); DBS-D: DBS stored at 4 °C for 4 months (T_4m_); DBS-E: DBS stored at room temperature (RT) for 4 months (T_4m_).

## Data Availability

The data presented in this study are openly available in the Harvard Dataverse https://dataverse.harvard.edu/dataset.xhtml?persistentId=doi:10.7910/DVN/DSHQ66 (accessed on 12 April 2024).
